# Studying the formation of the zone of calcified cartilage in bovine cartilage explants *ex vivo*^☆^

**DOI:** 10.1016/j.ocarto.2026.100743

**Published:** 2026-01-12

**Authors:** Jietao Xu, Andrea Schwab, Elias Salzer, Nicole Kops, Pieter A.J. Brama, Eric Farrell, Gerjo J.V.M. van Osch

**Affiliations:** a Department of Orthopedics and Sports Medicine, Erasmus MC, University Medical Center Rotterdam, Rotterdam, Netherlands; b Department of Orthopedics, Zhejiang Provincial People's Hospital, Hangzhou Medical College People's Hospital, Hangzhou, Zhejiang, China; c Experimental Orthopedics, University Orthopaedic Clinic, Otto-von-Guericke-University Magdeburg, Medical Faculty, Magdeburg, Germany; d School of Veterinary Medicine, University College Dublin, Dublin, Ireland; e Department of Oral and Maxillofacial Surgery, Erasmus MC, University Medical Center Rotterdam, Rotterdam, Netherlands; f Department of Otorhinolaryngology, Head and Neck Surgery, Erasmus MC, University Medical Center Rotterdam, Rotterdam, Netherlands; g Department of Biomechanical Engineering, Delft University of Technology, Delft, Netherlands

**Keywords:** Articular cartilage, Calcification, Zone of calcified cartilage, Tissue engineering, *ex vivo* culture model

## Abstract

**Objective:**

The zone of calcified cartilage (ZCC) connects non-calcified articular cartilage to the subchondral bone, acting as transitional layer. Regeneration of this layer is key for cartilage repair but remains a challenge. Knowledge on the formation of this layer during development is limited. This study describes the use of an *ex vivo* explant culture model to investigate the formation of the ZCC.

**Design:**

Explants were harvested from immature bovine metacarpophalangeal joints and cultured in the presence of β-glycerophosphate for 3 weeks as osteochondral explants, full-thickness cartilage or divided in top and bottom cartilage layers. To investigate cell-driven vs matrix-dependent calcification, explants were devitalized. Calcification was analysed using calcium uptake, micro-computed tomography, gene expression analysis, and histological stainings.

**Results:**

A distinct area of calcified cartilage formed in the explants *ex vivo.* This layer showed similar characteristics to the ZCC in mature bovine tissue. Viable chondrocytes in bottom layers actively contributed to cartilage calcification, while calcification in top layers was only present in devitalized top layer explants. Top layers inhibited cartilage calcification in bottom layers and expressed higher levels of *FGF18*, *PTHLH* and *MGP*, while the bottom layers expressed more *ALPL*, *COL10A1* and *IHH*.

**Conclusion:**

We present the first *ex vivo* model allowing to study and modulate cartilage calcification and the formation of the ZCC. We demonstrated an inherent zone-specific calcification pattern within the cartilage explants. This model allows future studies investigating mechanisms of ZCC formation in cartilage repair procedures, and the role of the top layer in pathological cartilage calcifications and potential interventions.

## 1 Introduction

Osteochondral defects caused by trauma or osteoarthritis lead to joint pain, dysfunction and reduced mobility [1]. The limited regenerative capacity of articular cartilage has stimulated the investigation of tissue engineering approaches. However, regenerating the zonal structure of this unit, especially the zone of calcified cartilage (ZCC), remains a major challenge [2]. Articular cartilage and subchondral bone are connected via the ZCC. This interfacial layer forms during skeletal maturation [[3], [4], [5], [6]]. The ZCC consists of dispersed chondrocytes residing in a physiological calcified cartilage like matrix [7]. As an interfacial layer, the ZCC ensures a mechanically continuous transition between the rigid and stiff bone and the viscoelastic and compliant articular cartilage [[8], [9], [10], [11]]. The tidemark defines the transition of the ZCC towards the non-calcified articular cartilage, while the cement line marks the transition to the subchondral bone [8]. The thin interfacial ZCC layer might play a role in cartilage and bone homeostasis and allows for molecular transport between the two tissues [12]. Therefore, regenerating the ZCC in osteochondral defects is critical for restoring physiological functions.

Understanding the mechanism underlying cartilage calcification is essential for the successful ZCC regeneration in (osteo-) chondral tissue engineering. The formation of the ZCC involves a multifactorial process resulting from a balance between pro-calcification factors and calcification inhibitors including cell-driven and matrix-dependent processes [13]. The physiological or pathological imbalances can be attributed to various factors, including chondrocyte phenotype, dysregulated inorganic pyrophosphate, inorganic phosphate metabolism and extracellular calcium levels [14].

*In vitro* 2D or 3D cell cultures are commonly used to study bone calcification processes. These models use either cell lines or chondrocytes isolated from immature cartilage [[15], [16], [17], [18], [19], [20]]. Conventional *in vitro* cell models do not include the extracellular matrix (ECM) nor the complex interplay between different cartilage zones. For instance, parathyroid hormone-related protein (PTHrP) in the superficial zone and Indian hedgehog signalling in the deep zone regulate cartilage calcification [21,22]. Although tissue engineering techniques have led to the generation of 3D culture models, the ECM produced in these models is immature and lacks the architecture of articular cartilage [[18], [19], [20]]. In contrast, *ex vivo* tissue cultures provide a more realistic environment maintaining cells in their native ECM [23,24]. Their utilisation has the potential to reduce the need for animal experiments in future research endeavours, aligning with ethical considerations. Obtaining healthy and immature human explants where the ZCC is not yet developed would be favorable. However, setting up such a cartilage calcification model is very challenging and raises ethical considerations as the ZCC forms during late childhood to adolescence.

Previously, the formation of the ZCC was investigated by ectopic implantation of bovine osteochondral explants *in vivo* in mice [25]. However, there are currently no explant models to study the formation of the ZCC *ex vivo*. In this study we developed a new *ex vivo* culture model to investigate the mechanism of the ZCC formation. We hypothesised that an explant of immature bovine cartilage (with the ZCC not yet formed) retains the ability to form the ZCC *ex vivo*. Then, we investigated the role of zonal cartilage and chondrocytes in calcified cartilage formation with this *ex vivo* cartilage culture model.

## 2 Materials and methods

### 2.1 Explant isolation and *ex vivo* culture

**Fig. 1:**
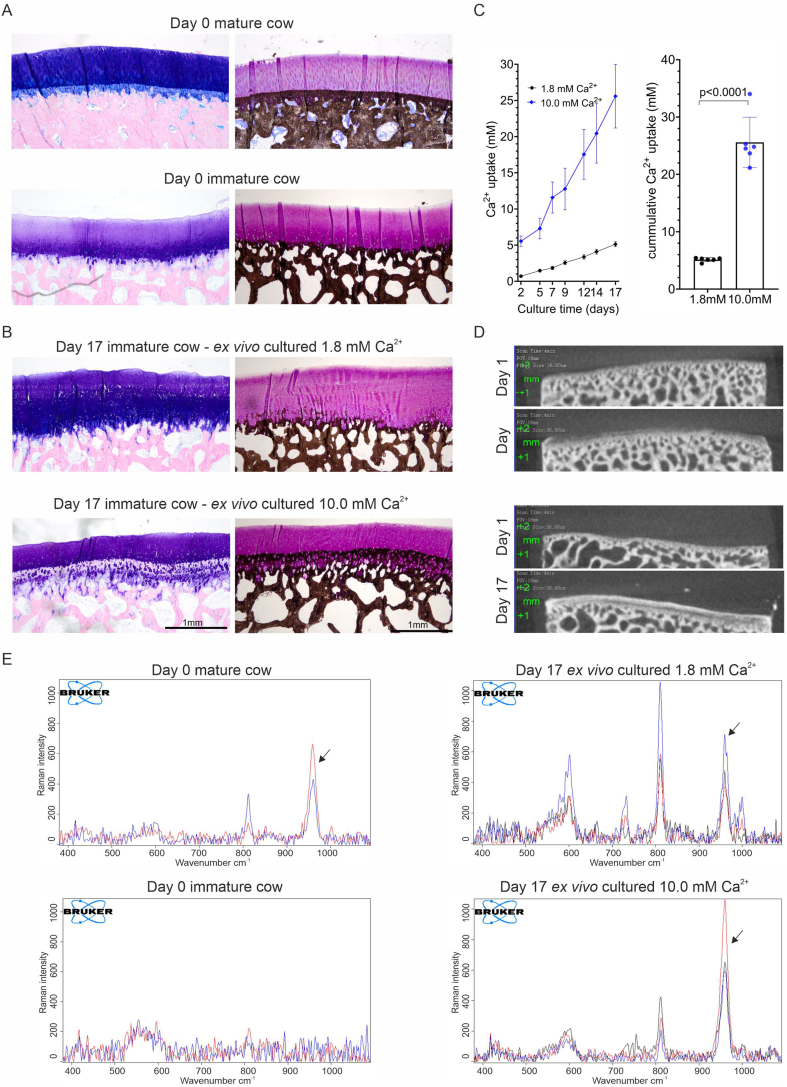
**A distinct layer of calcified cartilage was formed in articular cartilage in immature osteochondral explants *ex vivo*.** Histological staining of osteochondral explants derived from the metacarpophalangeal joints of (A) mature cows (2–3 years old), and (B) immature (6–8 months old) cows. The latter were either left uncultured (day 0) or cultured for 3 weeks with bGP (10 mM) and standard (1.8 mM) or high (10 mM) calcium. Giemsa-stained explants show the transition from articular cartilage (blue/purple) towards the zone of calcified cartilage (less intense staining) and the subchondral bone (pink). Von Kossa stain calcified tissue is stained black and glycosaminoglycans are stained purple (thionine). The scale bars indicate 1 mm. (C) Cumulative calcium uptake by the explants from the medium at each time point of medium refreshing during culture in media with high and standard calcium concentration (n = 6). Cummulative calcium uptake after 17 days of culture was higher in medium with high calcium concentration (10 mM) compared to standard concentration (1.8 mM). (D) Representative μCT images taken on day 1 and day 17 of the same explant that were cultured in standard and high calcium media confirm the formation of a zone of calcified cartilage *ex vivo*, with more evident calcifications in the high calcium condition. Unpaired *t*-test, n = 6 samples per condition. E) Raman spectra of representative samples harvested from the immature cow, mature cow and *ex vivo* cultured immature cow at high and low calcium concentration. The calcification associated basic calcium phosphate peak at 960 cm^−1^ was present in all samples, except for the immature cow, confirming the formation of calcium-phosphate deposits in the ECM of the cartilage at the cartilage-bone interface.

Full-thickness cartilage explants (4-mm diameter) and osteochondral explants (8-mm diameter and 5-mm height) were harvested from metacarpophalangeal joints of 6-8-month-old calves. All tissue used in this study was harvested from joints from a slaughterhouse (LifeTec Group, Eindhoven, The Netherlands) after the animals were killed for meat consumption in accordance with local regulations and this study does not involve animals. At the age of 6–8 months, the ZCC was not yet formed (Fig. 1, uncultured (day 0)). Explants were transferred to a well plate with Dulbecco's Modified Eagle Medium (DMEM, 1 g/L glucose, Gibco, Whatman, MA, USA) supplemented with 10 % fetal bovine serum (Gibco), 25 μg/mL ascorbic acid (Sigma-Aldrich, St. Louis, MO, USA), 50 μg/mL gentamycin (Gibco), and 1.5 μg/mL Amphotericin B (Gibco) and kept over-night at 37 °C and 5 % CO_2_. The next day, the cartilage explants were scanned with micro-computed tomography (μCT) and explants with remaining underlying subchondral bone tissue were excluded from the culture experiments. Explants were randomly divided into the respective experimental groups. To induce calcification, cartilage explants were cultured in a standard 24-well plate (1 mL per well). Osteochondral explants were transferred in a custom made well plate (LifeTec) designed to restrict the crosstalk between the tissue to the cartilage-bone interface (3 mL for bone and 3 mL for cartilage) [23]. Cartilage calcification was stimulated by media supplementation with 10 mM β-glycerophosphate (bGP, Sigma-Aldrich) for three weeks with media changes every 2–3 days. bGP serves a phosphate source in our culture media and forms calcium-phosphate deposits with free calcium ions available in the cultured sample. Osteochondral explants were additionally stimulated with elevated levels of calcium (10 mM) by the addition of calcium chloride (2 M stock in ultrapure water, Sigma-Aldrich) to the bGP containing media.

To study the inhibitory effect of the top cartilage layer on cartilage calcification, full-thickness cartilage explants were cut in half, creating two layers (referred to as top and bottom layer). Experiments for direct co-culture of top layers with a bottom layer were performed using a transwell system (PET membrane; 8 μm pore size, Life Sciences, Arizona, USA) in bGP containing media. Cartilage top and bottom layers were also used to investigate the effect of PTHrP (0–100 nM, Phoenix Pharmaceuticals, Burlingame, United States). Matrix associated cartilage calcification was studied on devitalized (4 % formalin treated for 3 h, Roth, Karlsruhe, Germany) cartilage explants (full thickness, top layer and bottom layer) cultured in bGP supplemented media in 24-well plates. After 3 weeks *ex vivo* culture, osteochondral and cartilage explants were formalin fixed (4 % formalin, 3 days) for histological analysis.

### 2.2 Calcium assay of the medium

Calcium concentration was measured in the medium harvested at every medium change and the cumulative calcium uptake was calculated. Calcium concentration in synovial fluid (n = 4, 6–8 months old calves) was measured as reference sample. 100 μl fresh reagent (0.5 M Ethanolamine, 0.175 mM o-cresolphthalein complexone, 9.9 mM 8-hydroxyquinoline, 0.3 M hydrochloric acid, Sigma-Aldrich) was added to 10 μl collected medium. Calcium chloride was used as standard to calculate the calcium concentration (absorbance measured at 570 nm, Versamax). Control medium without explants was measured to calculate the in calcium concentration, representing the amount of calcium taken up by the explants.

### 2.3 Micro-computed tomography (μCT) and quantification

Explants were scanned in a μCT scanner (Quantum GX, PerkinElmer, Akron, OH, USA) in medium (energy 90 KV, intensity 88–90 μA, FOV 172 mm (cartilage) and 36 mm (osteochondral), 86 μm isotropic voxel size, scan time 4 min). All scans were done under an X-ray filter of Cu (thickness = 0.06 mm) and Al (thickness = 0.5 mm). To quantify the volume of calcified tissue in cartilage explants, a single threshold was set. In each 3D reconstructed explant, the volume of interest was selected manually, and the calcification volumes were measured.

### 2.4 Gene expression analysis

The gene expression levels of fibroblast growth factor 18 (*FGF18*), parathyroid hormone-like hormone (*PTHLH*), matrix Gla protein (*MGP*), *ALPL*, collagen type X alpha 1 (*COL10A1*) and Indian hedgehog (*IHH*) of cartilage explants were assessed with Real-Time semiquantitative PCR (qPCR, primer details Supplementary Table 1.). Total RNA was extracted from cartilage explants harvested at day 0 following the RNA STAT-60 (Gentaur, Eersel, Belgium)/chloroform method until the harvesting of the aqueous phase, and reverse transcribed (RevertAid First Strand cDNA Synthesis Kit, Thermo Fisher Scientific, Massachusetts, US) following the manufacturer's instructions. SYBR green PCR kit (Eurogentec, Liege, Belgium) was used for qPCR. The protocol included a denaturation cycle at 95 °C for 10 min, 40 cycles of amplification (95 °C for 15 s, 60 °C for 60 s), and a melting curve analysis to check for amplified specificity. Relative expression was calculated using the Livak method (2^−ΔΔCt^), with Glyceraldehyde-3-Phosphate Dehydrogenase (*GAPDH*) as the reference gene.

### 2.5 Histological stainings

The formalin fixed cartilage explants were embedded in paraffin and sectioned (6 μm). For the histological characterization of the cartilage-bone interface in skeletal immature (6–8 months old cows) and skeletal mature (2–3 year old cows) tissue we embedded formalin fixed osteochondral tissue from MCP joints and osteochondral explants in methyl methacrylate (MMA) and sectioned (10 μm). Following dewaxing or MMA-removal, von Kossa/thionine staining was performed with 5 % silver nitrate solution (Sigma-Aldrich) and 0.4 % thionine (Sigma-Aldrich) to visualize calcium deposition in the extracellular matrix and cell/tissue morphology. To visualize the transition of the *ex vivo* stimulated calcified cartilage to the non-calcified cartilage, a Giemsa staining was performed. MMA sections were deplasticized and brought to water before immersion in Giemsa solution (Sigma-Aldrich) for 5 h. Sections were then dehydrated and mounted in Depex.

### 2.6 Raman spectroscopy

Confocal Raman spectroscopy (Bruker Senterra II under OPUS 7.8, Bruker, Karlsruhe, Germany) was performed on MMA tissue sections to characterize the calcium-phosphate type in the zone of calcified cartilage. Slides were deplastified, air dried and Raman spectra were recorded (785 nm laser, 100 mW, 10 coadditions, 5 s exposure, 20× objective). Raman spectra were recorded at the cartilage-bone interface. All spectra were cut (380 - 1090 cm^−1^) and baseline corrected (9 iterations) to correct for autofluorescence.

### 2.7 Statistical analysis

Statistical tests were performed using SPSS software 28.0 (SPSS Inc., Chicago, IL, USA). Normality was tested by a Shapiro-Wilk test. The homogeneity of variances was tested using Levene's test. Statistically significant differences between two groups were determined by a Student's T test or a Mann-Whitney *U* test. A one-way ANOVA or the Kruskal-Wallis H test with Bonferroni-correction were used to determine the statistically significant differences among three groups. A p-value ≤0.05 was considered statistically significant. The calcium concentration and volume were expressed as mean ± standard deviation (SD).

## 3 Results

### 3.1 Inherent zone-specific calcification and the formation of the ZCC in bovine osteochondral explants cultured in standard medium

Inherent zone-specific cartilage calcification was found in both standard (1.8 mM) and high calcium (10.0 mM) media conditions and limited to the lower part of the articular cartilage facing towards bone as shown by Giemsa staining and von Kossa-thionine staining (Fig. 1A and B and Supplementary Fig. 4). In the high calcium media, explants took up more calcium at each media change compared to the standard media with 1.8 mM calcium (Fig. 1C). μCT imaging (Fig. 1D) and von Kossa-thionine staining showed more calcified tissue formed in the high calcium compared to the standard calcium group. In both conditions, a region of non-calcified cartilage with mineral deposits was visible between this calcified area and the subchondral bone. Interestingly, a tidemark seemed to be formed at the upper end of the calcifying area in the osteochondral explants. The morphology of this upper interface of the ZCC formed in the *ex vivo* explants (10.0 mM) appeared similar to the tidemark in mature cows (Fig. 1A and B). Although the increase in calcium concentration in the media resulted in an increase in cartilage calcification, this condition is non-physiological and might lead to unphysiological calcifications. The standard calcium concentration in media (1.8 mM) mimics the calcium concentration in synovial fluid of 6–8 months old calves (1.46 ± 0.43 mM). Therefore, further experiments with cartilage explants were performed with the standard calcium concentration (1.8 mM) and medium exchanges every 2 days.

Hydroxyapatite is the most common form of basic calcium phosphate (BCP) crystals with a characteristic Raman peak at 960 cm^−1^ (Fig. 1E). The ZCC of the mature cows showed the BCP peak. The cartilage-bone interface of the immature calves did not show the BCP characteristic peak, thus confirming no calcified cartilage yet being formed. The Raman spectra of the ZCC that was formed *ex vivo* in the immature osteochondral samples showed the BCP peak associated with the deposition of hydroxyapatite indicating the formation of calcium-phosphate deposits in the ECM of the cartilage at the cartilage-bone interface. The sample specific location where the spectra were recorded are shown in Supplementary Fig. 3.

### 3.2 Cartilage calcification in bovine articular cartilage explants is restricted to the bottom layer transitioning towards the subchondral bone

**Fig. 2:**
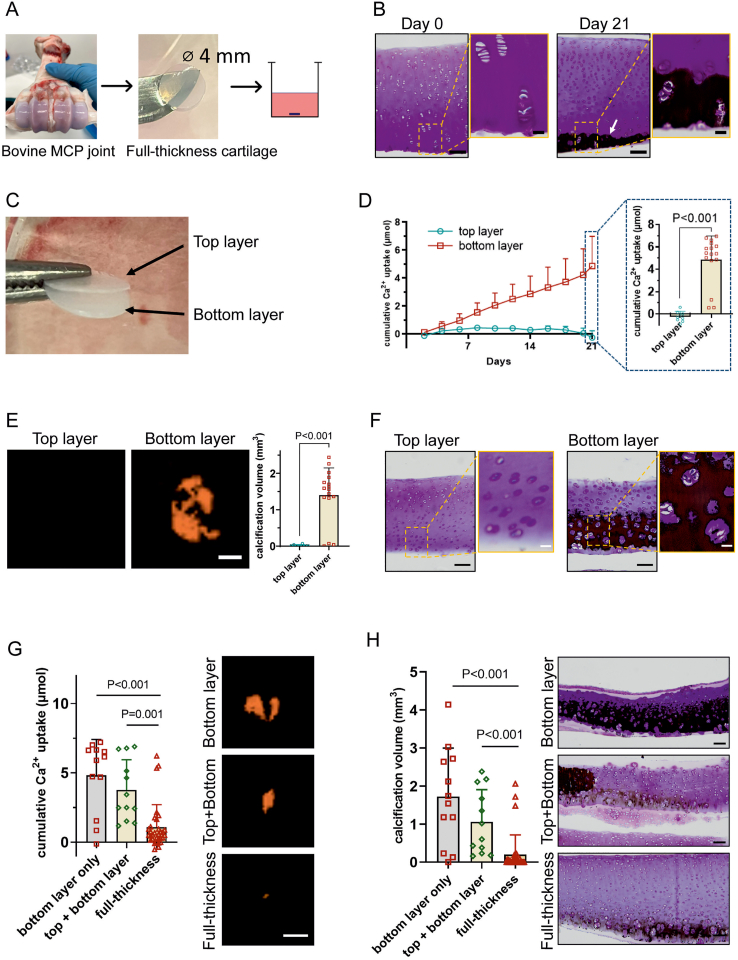
**Cartilage calcification in bovine articular cartilage explants occurs in the deep layer.** (A) Schematic for full-thickness cartilage explant harvest and culture set-up. (B) Representative von Kossa/thionine staining of full-thickness cartilage at day 0 and after 3 weeks in culture. The arrow indicates calcification indicated by von Kossa positive staining after 21 days (black). The scale bars are100 μm and 20 μm (magnified images), respectively. (C) Preparation of the top layer and the bottom layer explants from full thickness cartilage tissue. (D) The cumulative calcium (Ca^2+^) taken up from the medium by top layers (n = 8) or bottom layers (n = 16) of cartilage at each time point of medium refreshing during culture. The bar graph expresses the cumulative Ca^2+^ uptake at 3 weeks. Calcium uptake was higher in bottom layers. (E) Representative 3D reconstructed μCT images and volume of calcified tissue (mm^3^) after 3 weeks shows that calcification is almost exclusively found in bottom layers. Scale bar indicates 2 mm. (F) Representative von Kossa/thionine staining of a separately cultured top layer and a bottom layer after 3 weeks. While bottom layers show clear evidence of calcifications, top layers did not calcify. Scale bars indicate 100 μm and 20 μm (magnified images), respectively. (G) Cumulative Ca^2+^ taken up from the medium by different cartilage explants (bottom layer-only, n = 12; top + bottom layer with separated top layer and bottom layer co-cultured in the same well, n = 12; full-thickness cartilage, n = 32) and 3D reconstructed μCT images after 3 weeks stimulation with bGP. The scale bar indicates 2 mm. (H) Volume of calcified tissue (mm^3^) on μCT and representative von Kossa/thionine staining of cartilage explants cultured for 3 weeks. The scale bars indicate 100 μm. The Kruskal-Wallis H test was used to test statistical significance.

To study whether cartilage calcification is also possible in cartilage explants without the underlying subchondral bone in place, full thickness cartilage explants were cultured for 3 weeks in bGP containing media (Fig. 2A). Similar to the osteochondral explants, cartilage calcification in full thickness cartilage explants was limited to the deep layer (Fig. 2B). Interestingly, when explants were divided into top and bottom layers (Fig. 2C) and co-cultured, cumulative calcium uptake (Fig. 2D) and μCT derived calcification volume (Fig. 2E–G) were higher in bottom layers compared to full thickness and top layer only explants. This finding correlated with the von Kossa staining showing more calcification in the bottom only layer explants (Fig. 2F–H). Calcification was exclusively present in the bottom layer of full thickness and bottom layer explants. No calcification was observed in the top layers of any condition.

### 3.3 Viable chondrocytes in articular cartilage explants modulate cartilage calcification zone-dependently

**Fig. 3:**
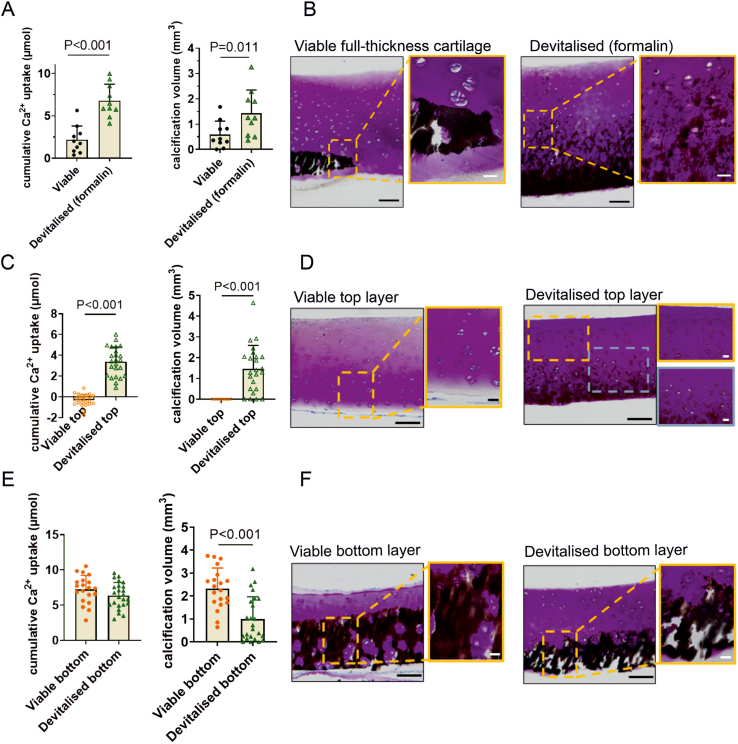
**Viable chondrocytes in full-thickness cartilage modulate cartilage calcification zone dependently.** Cartilage explants were cultured for 3 weeks in bGP media. (A) Cumulative calcium (Ca^2+^) uptake (n = 10) by viable and devitalized full-thickness cartilage explants, the volume of calcified tissue (mm^3^) and (B) Representative von Kossa/thionine staining of cartilage explants. Calcium uptake was higher in devitalized explants. The scale bars indicate 100 μm and 20 μm (magnified images), respectively. (C) Cumulative Ca^2+^ uptake by viable top layers (n = 23) and devitalized top layers (n = 24), volume of calcified tissue (mm^3^) and (D) representative von Kossa/thionine staining of cartilage explants. Top layers showed calcification after devitalization. The scale bars indicate 100 μm and 20 μm (magnified). (E) Cumulative Ca^2+^ uptake by viable bottom layers (n = 20) and devitalized bottom layers (n = 24) and the volume of calcified tissue (mm^3^) and (F) representative von Kossa/thionine staining of cartilage explants. Viable bottom layers showed more calcification than devitalized explants. The scale bars indicate 100 μm and 20 μm (magnified images), respectively. The Mann-Whitney *U* test was used to test statistical significance.

Devitalization of chondrocytes in explants was confirmed in live-dead staining (Supplementary Fig. 1). In full thickness cartilage explants, more calcification was observed in devitalized explants compared to viable explants with an increased cumulative calcium uptake and calcification volume (Fig. 3A). Von Kossa/thionine staining showed calcification in both groups in the deep zone (Fig. 3B). Interestingly, devitalized top layer explants showed cartilage calcification while in viable top layers no signs of calcification were observed (Fig. 3C and D). In contrast, devitalized bottom layers showed less calcification than viable bottom layers based on calcium uptake and von Kossa/thionine staining (Fig. 3E and F). This suggests that the chondrocytes in the explants actively contribute to cartilage calcification in our model.

### 3.4 Cartilage calcification can be modulated by the top layer cartilage explants

**Fig. 4:**
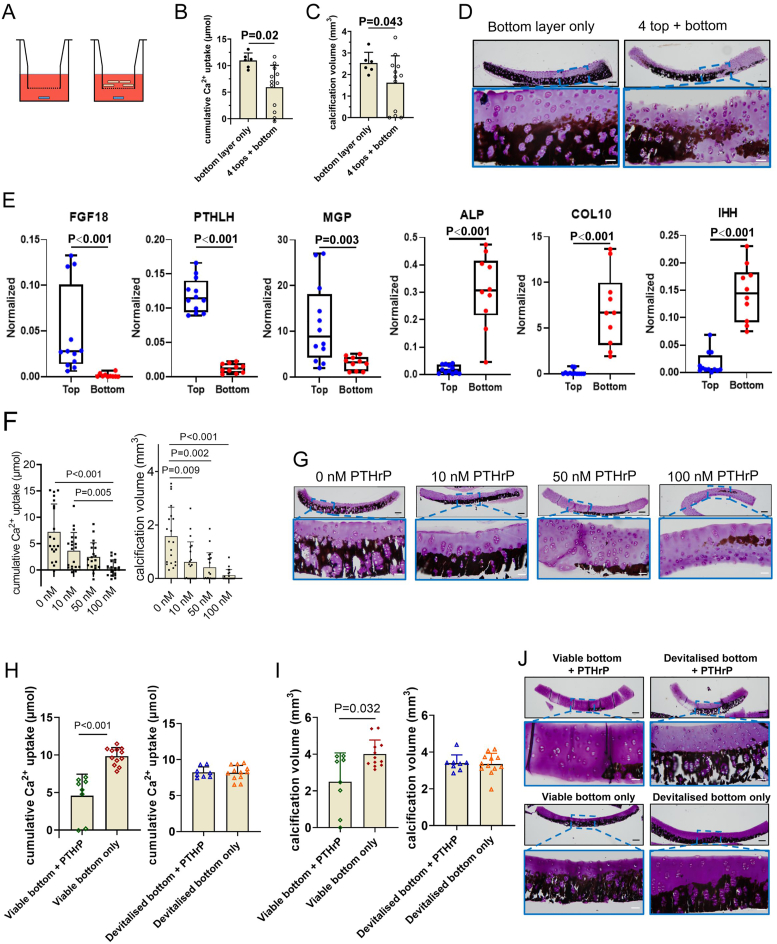
**Modulation of *ex vivo* cartilage calcification is a result of PTHLH expressed by chondrocytes in the top layers.** Explants were cultured for 3 weeks in bGP media. (A) Schematic illustration of the indirect co-culture of four top layers (beige) with a single bottom layer (blue) in a transwell system. (B) Cumulative calcium (Ca^2+^) taken up from the medium by cartilage explants (bottom layer-only, n = 6; 4 top layers + 1 bottom layer, n = 12), (C) volume of calcified tissue (mm^3^) and (D) representative von Kossa/thionine staining of cartilage explants. Calcification in bottom layers were reduced in the co-culture system with 4 top layers compared to bottom layer only. The scale bars indicate 200 μm and 50 μm (magnified images), respectively. (E) Relative gene expressions of FGF18, PTHLH, MGP, ALPL, COL10A1 and Indian hedgehog in chondrocytes residing in fresh top layers and bottom layers harvested from young (6–8 months old) calves. The top layers have a higher expression of genes associated to calcification-inhibition while in the bottom layers genes associated to calcification are higher expressed. (F) Cumulative Ca^2+^ uptake of cartilage explants (n = 20) and calcification volume cultured with 0–100 nM PTHrP to inhibit calcification. (G) Representative von Kossa/thionine staining of cartilage explants. Calcification inhibition was dose-dependent. The yellow squares indicate the magnified images. The scale bar indicates 200 μm and 50 μm (magnified images), respectively. (H) Viable and devitalized bottom layer explants were cultured with or without 100 nM PTHrP. (H) Cumulative Ca^2+^ uptake, (I) the volume of calcified tissue (mm^3^), and (J) representative von Kossa/thionine staining of cartilage explants. Calcifications were only inhibited by PTHrP in viable bottom layers, not in devitalized bottom layers. The scale bar indicates 200 μm and 50 μm (magnified images), respectively.

With the knowledge that there was no calcification present in viable top cartilage layers, we hypothesised that the presence of bioactive factors originating from the top layer potentially inhibit cartilage calcification. Therefore, we co-cultured bottom layers with four top layers in a transwell system (Fig. 4A). Cartilage calcification was reduced in bottom layers co-cultured with top layers based on cumulative calcium uptake, calcification volume (Fig. 4B and C). Von Kossa/thionine staining showed a similar trend (Fig. 4D). The calcification in the devitalized bottom layers compared to viable bottom layers was even further reduced when co-cultured with viable top layers (Supplementary Fig. 2A–B), but also with devitalized top layers (Supplementary Fig. 2C–D). To investigate the zonal differences between bottom and top cartilage layer in more detail, gene expression analysis of non-cultured explants was performed. Chondrocytes in top layers expressed higher levels of the calcification-inhibiting factors *FGF18*, *PTHLH* and *MGP*. Conversely, chondrocytes in the bottom layers exhibited elevated expression of *ALPL*, *COL10A1* and *IHH*, which were hardly expressed in top layers and are associated with cartilage calcification and chondrocyte hypertrophy (Fig. 4E).

*PTHLH* was the most differently expressed gene comparing the top and bottom cartilage layers. Therefore, we selected this peptide (PTHrP) to screen its calcification inhibitory effect on bottom layer explants at concentrations ranging between 0 and 100 nM. A dose-dependent reduction in calcium uptake, calcification volume and von Kossa/thionine staining was observed with 100 nM PTHrP showing the strongest inhibitory effect (Fig. 4F and G). Additionally, the addition of PTHrP (100 nM) inhibited calcification in viable bottom layers but not in devitalized explants (Fig. 4H–J).

## 4 Discussion

In this study, we developed an *ex vivo* culture model to investigate the formation of the ZCC in articular cartilage. Cartilage calcification at the cartilage-bone interface is a physiological process taking place during skeletal maturation and results in the formation of the ZCC [[3], [4], [5], [6]]. In experimental set ups ranging from osteochondral explants to cartilage explant top and bottom layers we showed a zone-specific calcification restricted to the bottom layer of viable articular cartilage. We showed that the cartilage top layer can undergo calcification once it is devitalized, highlighting the active inhibition of calcification in this layer by chondrocytes in the top layer. Moreover, the top layer demonstrated the capacity to regulate the calcification in the bottom layer by secreted factors. In contrast, the bottom layer calcifies irrespective of cell viability, albeit viable cells actively increase calcification. To the best of our knowledge, this is the first *ex vivo* model investigating the formation of the ZCC and how cartilage calcification can be modulated.

*Ex vivo* explant culture models represent powerful tools in cartilage research, bridging the gap between *in vitro* models and pre-clinical studies and reducing the need for animal experiments in our research endeavours [[26], [27], [28]]. Our bovine explant culture model includes the different layers of articular cartilage that contain chondrocytes of different phenotypes and ECM of different composition [29]. The presence of an ECM prevents the de-differentiation that often occurs in chondrocyte monolayer cultures. By retaining the zonal organisation and 3D structure, the specific biochemical gradients and chondrocyte phenotypes present within each cartilage zone can be preserved. Osteochondral and cartilage explants from skeletally immature metacarpophalangeal joints formed a distinct calcified layer in the articular cartilage at the cartilage-bone interface *ex vivo.* This layer resembles the ZCC in skeletally mature bovine metacarpophalangeal joints. We propose that the calcified cartilage formed in the *ex vivo* model is linked to the formation of the ZCC. This assumption is supported by first indications of the formation of the tidemark at the upper end of the non-calcified cartilage together with the cement line at the interface to the bone based on histological stainings (Supplementary Fig. 4). Since there are no markers known on cell phenotype or tissue matrix level that allow the differentiation of chondrocytes in the ZCC from the non-calcified cartilage, we are limited to histo-morphometric comparison. Comparing the histological stainings, the morphology, the low amplitude of the tidemark and the waviness and amplitude of the cement line in mature cows, we believe that the ZCC in the bovine *ex vivo* osteochondral explants indeed is the pre-mature ZCC [30,31]. One characteristic of the ZCC is to distribute loads between the soft non-calcified cartilage and the stiff subchondral bone. The biomechanical differences between calcified and non-calcified articular cartilage are up to 6-fold for the elastic modulus and up to 10-fold for stiffness for human articular cartilage [32,33]. However, little is known on bovine cartilage. In future studies, biomechanical studies should be implemented on the *ex vivo* cultured explants to further characterize the mechanical properties of the interface.

Depending on the research question the cartilage explants, osteochondral explants or top vs bottom cartilage cultures can be used to study in more detail mechanisms associated with cartilage calcification and potential treatments to improve cartilage and osteochondral unit regeneration in future studies. While osteochondral explants are necessary to study the interaction of cartilage with bone, cartilage explants are suitable to study specific inhibitors and/or promotors in cartilage calcification and to identify calcification-associated pathways.

Physiological calcium concentrations of 1.8 mM (standard concentration in DMEM media) together with 10 mM bGP were sufficient to stimulate cartilage calcification in the novel *ex vivo* model. This standard calcium concentration of DMEM is comparable to the calcium concentration in synovial fluid of 6–8 months old calves. Higher calcium concentrations were previously suggested to potentially stimulate cartilage maturation [34,35]. However, little is known about the interaction of the tissue and the surrounding medium in terms of calcium concentrations and the response on chondrocytes. As high calcium concentrations (10 mM) led to intense calcifications that might have been pathophysiological, standard calcium concentrations were preferred in our model in all following experiments. Faure *et al.* studied cartilage calcification in human tissues derived from osteoarthritis patients [36]. Comparing our bovine calcification model of immature cartilage to this mature human *ex vivo* cartilage calcification model, the bovine explant model resulted in a stronger response to bGP with a calcification volume in the range of mm^3^ compared to the very limited and locally appearing calcification on the superficial layer of less than 20 μg in the human model [36]. This implies a very different location and potentially different mechanism of calcification mainly explained by the maturity of the bovine (immature) and human (mature and diseased) cartilage explants. In this study, we also showed that there are calcification inhibitors present in the top layers of cartilage of developing and skeletally mature animals.

The difference in the expression of pro-calcifying factors and calcification inhibitors contribute to the control of cartilage calcification and the zonal calcification limited to the bottom area of immature bovine cartilage. When the top and bottom layer of the articular cartilage explants were separated, the bottom layer but not the top layer calcified *ex vivo*. To our surprise, in this set-up the calcification volume in the bottom layer explants was higher compared to the volume in full thickness explants. This does not only indicate that the cartilage tissue in the bottom layer is predestined to calcify, but importantly also that the presence of the top layer inhibits the calcification process in the bottom layer. We hypothesised that bioactive factors released by chondrocytes in the top layer inhibit calcification, while factors released by chondrocytes in the bottom layer stimulate calcification. To test this hypothesis, we co-cultured four top layers with one bottom layer, resulting in the reduction of cartilage calcification in the bottom layer. Our data suggest that chondrocytes residing in the top layers actively contribute to control cartilage calcification. These findings are in line with co-culture experiments of neonatal bovine chondrocytes isolated from the different zones showing an inhibitory effect of top layer chondrocytes on the calcification propensity of bottom layer derived chondrocytes in a direct co-culture set up [37]. Interestingly, top layer explants showed signs of calcification in devitalized top layer highlighting that the inhibitory effect on the cartilage top layer is dependent on viable chondrocytes in this layer. In contrast, devitalized explants of the cartilage top layer even inhibited cartilage calcification in explants of viable cartilage bottom layer. In summary, these data show that the top layer has an important role in regulating the formation of the ZCC in the deep layer of articular cartilage.

Tissue calcification is a result of an active cell-driven and a passive matrix-dependent process. In this study, we showed that viable chondrocytes in bottom layers actively contribute to cartilage calcification, while viable chondrocytes in the top layers are more prone to inhibit calcification. Interestingly, calcification in the cartilage top layer was only present in devitalized top layer explants. In contrast, bottom layer explants underwent calcification in explants with viable and in devitalized chondrocytes. One explanation for the zonal difference in cartilage calcification are the zonal differences in the gene expression of chondrocyte subpopulation. While cells in the top layers showed more dominant expression of chondrogenesis genes (*FGF18*) and genes associated with calcification inhibition (*PTHLH* and *MGP*)*,* chondrocytes in the bottom layer expressed more genes related to cartilage calcification or hypertrophy (*ALPL*, *COL10A1* and *IHH*) [21,22,38,39]. Notably, the calcium sensitive PTHrP pathway has been shown to be involved in chondrocyte maturation and matrix production as well, which was not investigated in this study [35,40]. Besides PTHLH, MGP has also been described to play a role in cartilage calcification acting as calcification inhibitor. Chondrocytes in the top layer expressed higher levels of *MGP* compared to cells in the bottom layer. MGP forms complexes with fetuin A that inhibit the crystallization of calcium phosphate and crystal growth and thus acting as calcification inhibitor [41]. In an *in vivo* study, mgp-deficient mice showed spontaneous calcification of growth plate cartilage and arteries [38]. In line with the explant co-culture experiment, the addition of exogenous PTHrP indeed inhibited calcifications in a dose-dependent manner. This suggests PTHrP as a candidate to inhibit cartilage calcification in immature cartilage explants. Other candidates are MGP and fetuin A that form complexes that inhibit the crystallization of calcium phosphate and crystal growth and thus acting as calcification inhibitor [41]. These genes have previously been found to be involved in cartilage and chondrocyte maturation, in particular in the growth plate and in osteoarthritis and our data indicate they play a role in formation of the ZCC as well.

This bovine *ex vivo* model can be used and modified to study mechanisms of the physiological calcification during the formation of the ZCC and thus to provide useful insights into treatments to improve the regeneration of cartilage or osteochondral unit. Cartilage calcification is orchestrated by the interplay of calcification inhibiting and stimulating factors produced by chondrocytes in different zones. We also demonstrated that the ability of cartilage to calcify depends on zonal differences. Additionally, the *ex vivo* model(s) offer a platform for testing novel compounds targeting calcification inhibitors as a potential therapeutic approach in calcification disorders during skeletal development as well as for testing novel approaches to improve osteochondral tissue regeneration. In future studies, our models could be advanced to study pathological calcification such as tidemark duplication and the advancement of the ZCC during osteoarthritis.

## Author contributions

Conception and design: JX, AS, ES, GJVMVO, EF, PB.

Collection and assembly of data: JX, AS, ES, NK.

Analysis and interpretation of the data: JX, AS, ES, NK, GJVMVO, EF.

Drafting of the article: JX, AS, ES, NK.

Critical revision of the article for important intellectual content: GJVMVO, EF, PB.

Final approval of the article: all.

Obtaining of funding: GJVMVO, EF, AS, JX.

Administrative, technical, or logistic support: NK.

## Declaration of Generative AI and AI-assisted technologies in the writing process

During the preparation of this work the authors did not use any AI or AI-assisted technologies.

## Declaration of competing of interest statement

The authors have no conflicts of interest to declare.

## References

[1] Deng C., Chang J., Wu C. (2019). Bioactive scaffolds for osteochondral regeneration. J. Orthop. Transl..

[2] Zhou L., Gjvm V.O., Malda J., Stoddart M.J., Lai Y., Richards R.G., Ki-wai Ho K., Qin L. (2020). Innovative tissue-engineered strategies for osteochondral defect repair and regeneration: current progress and challenges. Adv. Healthcare Mater..

[3] Evans L.A.E., Pitsillides A.A. (2022). Structural clues to articular calcified cartilage function: a descriptive review of this crucial interface tissue. J. Anat..

[4] Simkin P.A. (2008). A biography of the chondrocyte. Ann. Rheum. Dis..

[5] Kazemi M., Williams J.L. (2021). Properties of cartilage-subchondral bone junctions: a narrative review with specific focus on the growth plate. Cartilage.

[6] Bhosale A.M., Richardson J.B. (2008). Articular cartilage: structure, injuries and review of management. Br. Med. Bull..

[7] Zhou H., Yuan L., Xu Z., Yi X., Wu X., Mu C., Ge L., Li D. (2022). Mimicking the composition and structure of the osteochondral tissue to fabricate a heterogeneous three-layer scaffold for the repair of osteochondral defects. ACS Appl. Bio Mater..

[8] Broom N.D., Poole C.A. (1982). A functional-morphological study of the tidemark region of articular cartilage maintained in a non-viable physiological condition. J. Anat..

[9] Radin E.L., Rose R.M. (1986).

[10] Mente P.L., Lewis J.L. (1994). Elastic modulus of calcified cartilage is an order of magnitude less than that of subchondral bone. J. Orthop. Res..

[11] Oegema T.R., Carpenter R.J., Hofmeister F., Thompson R.C. (1997). The interaction of the zone of calcified cartilage and subchondral bone in osteoarthritis. Microsc. Res. Tech..

[12] Wang W., Ye R., Xie W., Zhang Y., An S., Li Y., Zhou Y. (2022). Roles of the calcified cartilage layer and its tissue engineering reconstruction in osteoarthritis treatment. Front. Bioeng. Biotechnol..

[13] Ea H.-K., Nguyen C., Bazin D., Bianchi A., Guicheux J., Reboul P., Daudon M., Lioté F. (2011). Articular cartilage calcification in osteoarthritis: insights into crystal-induced stress. Arthritis Rheum..

[14] Hamade T., Bianchi A., Sebillaud S., Netter P., Jouzeau J.Y., Cailotto F. (2010). Inorganic phosphate (Pi) modulates the expression of key regulatory proteins of the inorganic pyrophosphate (PPi) metabolism in TGF-β1-stimulated chondrocytes. Bio Med. Mater. Eng..

[15] Hinek A., Reiner A., Poole A.R. (1987). The calcification of cartilage matrix in chondrocyte culture: studies of the C-propeptide of type II collagen (chondrocalcin). J. Cell Biol..

[16] Kandel R., Boyle J., Gibson G., Cruz T., Speagle M. (1997). In vitro formation of mineralized cartilagenous tissue by articular chondrocytes. In Vitro Cellular & Develop. Biol Animal..

[17] Yan J., Shen M., Sui B., Lu W., Han X., Wan Q., Liu Y., Kang J., Qin W., Zhang Z., Chen D., Cao Y., Ying S., Tay F.R., Niu L.N., Jiao K. (2022). Autophagic LC3(+) calcified extracellular vesicles initiate cartilage calcification in osteoarthritis. Sci. Adv..

[18] Wang Y., Kim U.J., Blasioli D.J., Kim H.J., Kaplan D.L. (2005). In vitro cartilage tissue engineering with 3D porous aqueous-derived silk scaffolds and mesenchymal stem cells. Biomaterials.

[19] Little C.J., Bawolin N.K., Chen X. (2011). Mechanical properties of natural cartilage and tissue-engineered constructs. Tissue Eng., Part B.

[20] Chen J., Yuan Z., Liu Y., Zheng R., Dai Y., Tao R., Xia H., Liu H., Zhang Z., Zhang W., Liu W., Cao Y., Zhou G. (2017). Improvement of in vitro three-dimensional cartilage regeneration by a novel hydrostatic pressure bioreactor. Stem Cells Transl. Med..

[21] Tsukazaki T., Ohtsuru A., Enomoto H., Yano H., Motomura K., Ito M., Namba H., Iwasaki K., Yamashita S. (1995). Expression of parathyroid hormone-related protein in rat articular cartilage. Calcif. Tissue Int..

[22] Miao D., Scutt A. (2002). Histochemical localization of alkaline phosphatase activity in decalcified bone and cartilage. J. Histochem. Cytochem..

[23] Schwab A., Meeuwsen A., Ehlicke F., Hansmann J., Mulder L., Smits A., Walles H., Kock L. (2017). Ex vivo culture platform for assessment of cartilage repair treatment strategies. ALTEX.

[24] Salzer E., Schmitz T.C., Mouser V.H., Vernengo A., Gantenbein B., Jansen J.U., Neidlinger-Wilke C., Wilke H.J., Grad S., Le Maitre C.L., Tryfonidou M.A., Ito K. (2023). Ex vivo intervertebral disc cultures: degeneration-induction methods and their implications for clinical translation. Eur. Cell. Mater..

[25] Ng J., Wei Y., Zhou B., Bhumiratana S., Burapachaisri A., Guo E., Vunjak-Novakovic G. (2018). Ectopic implantation of juvenile osteochondral tissues recapitulates endochondral ossification. J. Tissue Eng. Regen. Med..

[26] Anderson J.R., Phelan M.M., Foddy L., Clegg P.D., Peffers M.J. (2020). Ex vivo equine cartilage explant osteoarthritis model: a metabolomics and proteomics study. J. Proteome Res..

[27] Monaco G., El Haj A.J., Alini M., Stoddart M.J. (2021). Ex vivo systems to study chondrogenic differentiation and cartilage integration. Journal of Functional Morphology and Kinesiology.

[28] Trengove A., Duchi S., Onofrillo C., Sooriyaaratchi D., Di Bella C., O'Connor A.J. (2024). Bridging bench to body: ex vivo models to understand articular cartilage repair. Curr. Opin. Biotechnol..

[29] Jeon J., Malda J., Schrobback K., Irawan D., Masuda K., Sah R.L., Hutmacher D.W., Klein T., Berthiaume F., Morgan J.R. (2010). Methods in Bioengineering: 3D Tissue Engineering.

[30] Thambyah A., Broom N. (2009). On new bone formation in the pre-osteoarthritic joint. Osteoarthr. Cartil..

[31] Thambyah A., Broom N. (2006). Micro-anatomical response of cartilage-on-bone to compression: mechanisms of deformation within and beyond the directly loaded matrix. J. Anat..

[32] Das Gupta S., Workman J., Finnilä M.A.J., Saarakkala S., Thambyah A. (2022). Subchondral bone plate thickness is associated with micromechanical and microstructural changes in the bovine patella osteochondral junction with different levels of cartilage degeneration. J. Mech. Behav. Biomed. Mater..

[33] Fan X., Wu X., Trevisan Franca De Lima L., Stehbens S., Punyadeera C., Webb R., Hamilton B., Ayyapann V., McLauchlan C., Crawford R., Zheng M., Xiao Y., Prasadam I. (2022). The deterioration of calcified cartilage integrity reflects the severity of osteoarthritis-A structural, molecular, and biochemical analysis. FASEB J..

[34] Amin A.K., Huntley J.S., Bush P.G., Simpson A.H., Hall A.C. (2009). Chondrocyte death in mechanically injured articular cartilage--the influence of extracellular calcium. J. Orthop. Res..

[35] Hammersen T., Buchert J., Zietzschmann S., Diederichs S., Richter W. (2023). Inverse regulation of cartilage neogenesis at physiologically relevant calcium conditions by human articular chondrocytes and mesenchymal stromal cells. Cells.

[36] Faure E., Wegrzyn J., Bernabei I., Falgayrac G., Bertheaume N., Pascart T., Hugle T., Busso N., Nasi S. (2025). A new ex vivo human model of osteoarthritis cartilage calcification. Rheumatology.

[37] Jiang J., Leong N.L., Mung J.C., Hidaka C., Lu H.H. (2008). Interaction between zonal populations of articular chondrocytes suppresses chondrocyte mineralization and this process is mediated by PTHrP. Osteoarthr. Cartil..

[38] Luo G., Ducy P., McKee M.D., Pinero G.J., Loyer E., Behringer R.R., Karsenty G. (1997). Spontaneous calcification of arteries and cartilage in mice lacking matrix GLA protein. Nature.

[39] Delve E., Bromand S., St-Pierre J.P., Grynpas M., Kandel R. (2015). Superficial zone chondrocytes secrete a factor that regulates deep zone cartilage mineralization by modulating polyphosphate levels. Osteoarthr. Cartil..

[40] Vortkamp A., Lee K., Lanske B., Segre G.V., Kronenberg H.M., Tabin C.J. (1996). Regulation of rate of cartilage differentiation by Indian hedgehog and PTH-related protein. Science.

[41] Wallin R., Schurgers L.J., Loeser R.F. (2010). Biosynthesis of the vitamin K-dependent matrix gla protein (MGP) in chondrocytes: a fetuin–MGP protein complex is assembled in vesicles shed from normal but not from osteoarthritic chondrocytes. Osteoarthr. Cartil..

