# Supplementary material for: Studying the formation of the zone of calcified cartilage in bovine cartilage explants ex vivo
Source: Osteoarthr Cartil Open. 2026 Jan 12;8(1):100743. doi: 10.1016/j.ocarto.2026.100743 (PMC12856866; doi:10.1016/j.ocarto.2026.100743)
Supplement: Multimedia component 1 [file mmc1.docx]

**SUPPLEMENTARY Information**

Table 1: List of primers and primer sequences.

| **Gene** | **Forward Primers** | **Reverse Primers** |
| --- | --- | --- |
| *GAPDH* | 5’-TTCAACGGCACAGTCAAGG-3’ | 5’-CATACTCAGCACCAGCATCAC-3’ |
| *FGF18* | 5’-AAGTCCGGATCAAGGGCAAG-3’ | 5’-CGACATCAGGGCTGTGTAGT-3’ |
| *PTHLH* | 5’-AAATAAGTCCCCAGAGCGAGA-3’ | 5’-TGCTCAGACACAGCCCTTTT-3’ |
| *MGP* | 5’-TCACGAAAGCCTGGAATCCT-3’ | 5’-TCCCGGTTGAGCTCGTATTG-3’ |
| *ALPL* | 5’-GTCTGGAACCGCACTGATCT-3’ | 5’-GAGTGAAGGGTCAGTCGCAT-3’ |
| *COL10A1* | 5’-ACTTCTCTTACCACATACACGTGAAAG-3’ | 5’-CCAGGTAGCCCTTGATGTACTCA-3’ |
| *IHH* | 5’-CGGCTTCGACTGGGTGTATT-3’ | 5’-TCACAGCTGACAAGGCCACA-3’ |

**
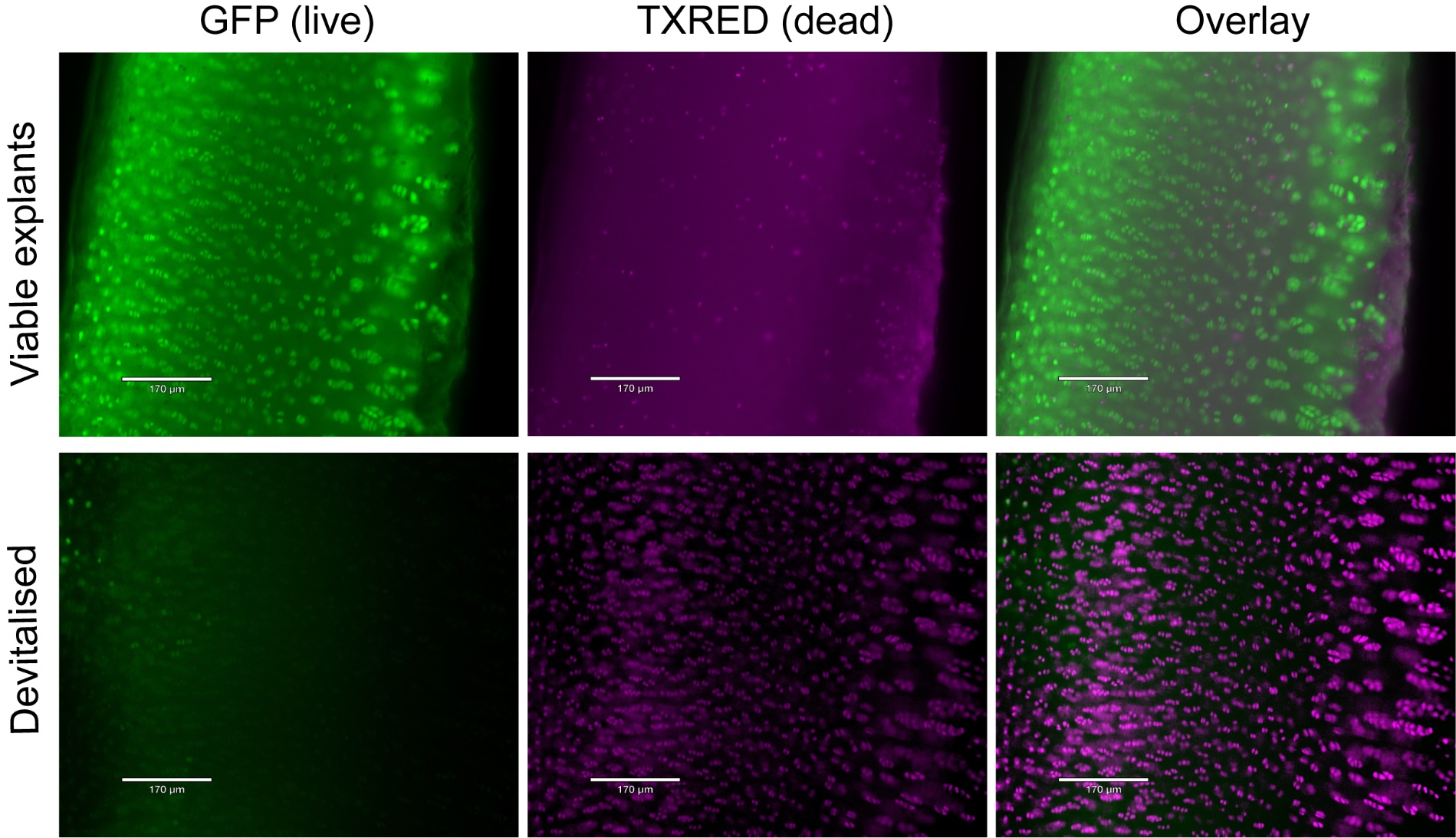
**

Supplementary Figure 1. Live-dead staining of native and devitalized resulting in the majority of dead chondrocytes (purple staining) in the devitalized cartilage explants using formalin treatment. In viable explants (no treatment) the majority of the cells were viable (green) with few dead cells throughout the explant. Scale bar 170 µm.


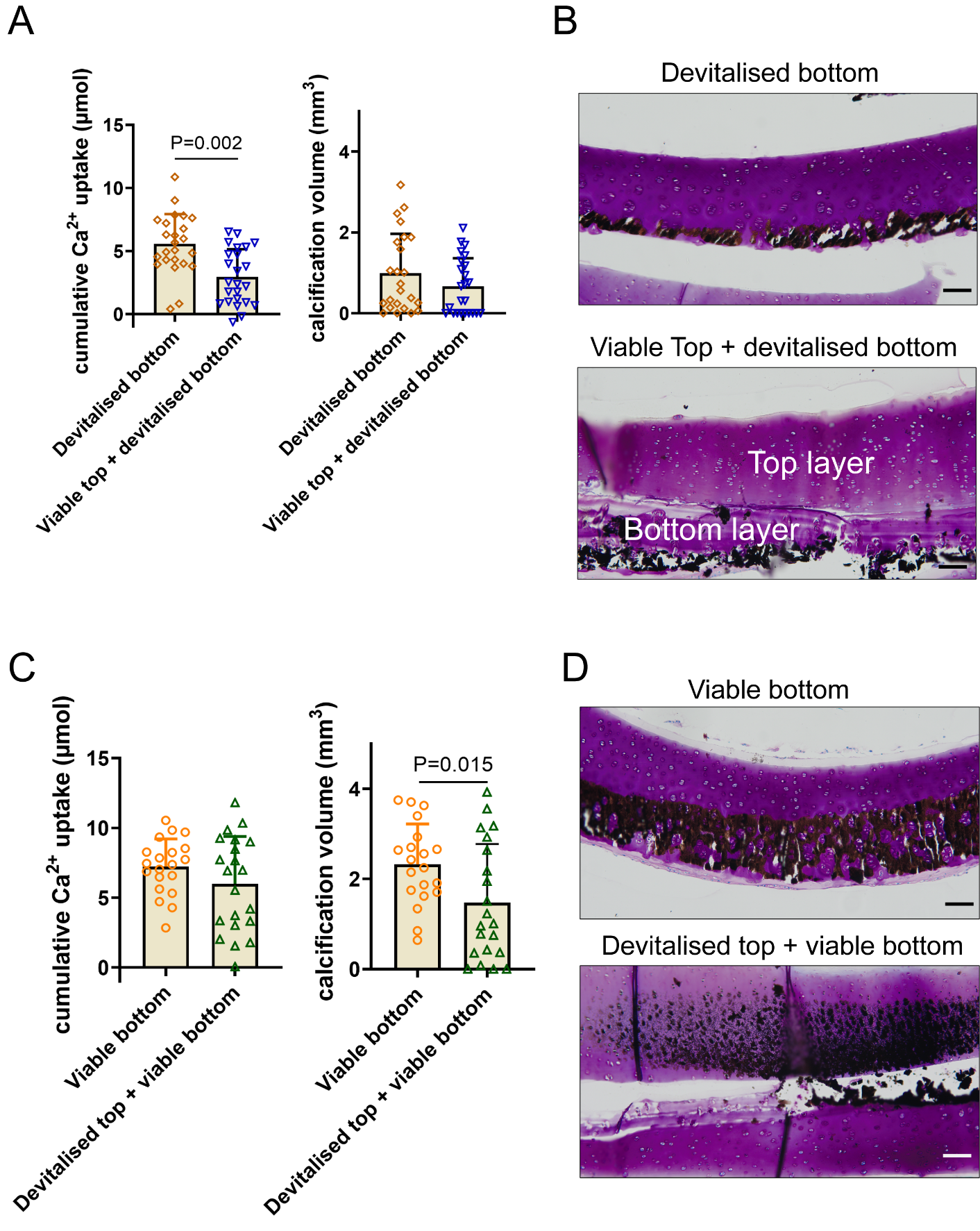


Supplementary Figure 2: The role of viable chondrocytes in the interplay between the top and bottom cartilage layers resulting in calcification in both layers after devitalization after 3 weeks. (A) Cummulative calcium uptake and calcification volume of devitalized bottom layer resulted in reduced calcification in bottom leayers when co-cultured with viable top layer. (B) Representative von Kossa/thionine staining of cartilage explants with von Kossa positive staining in bottom layers. The scale bars indicate 100 µm. (C) Cummulative calcium uptake and calcification volume of devitalized top layers and devitalized top layers with viable bottom layers. (D) Representative von Kossa/thionine staining with von Kossa positive staining in the bottom layers in both conditions. The scale bars indicate 100 µm. Scale bars 100 µm.


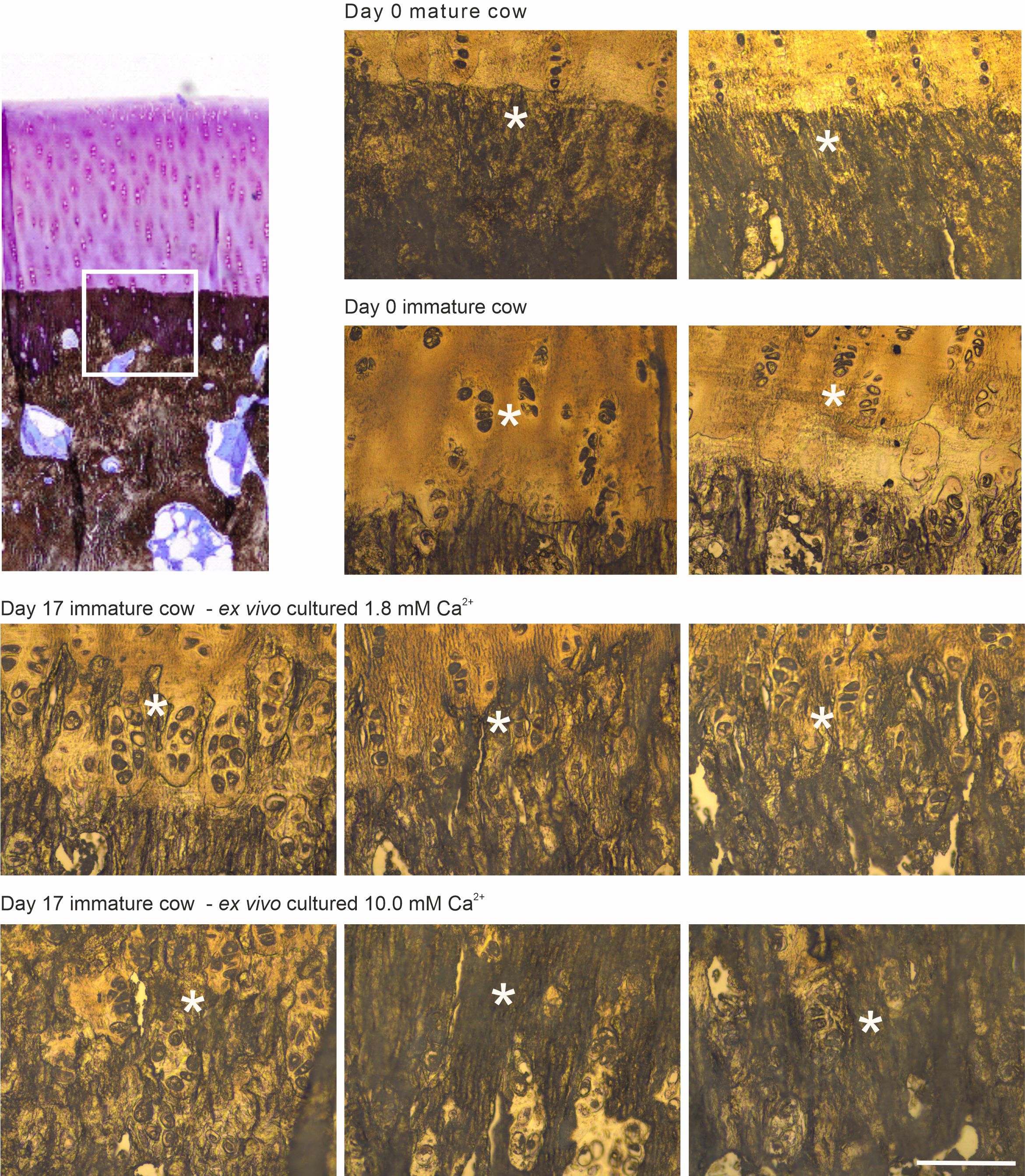


Supplementary Figure 3: Selection of the area of interest at the cartilage-bone interface to perform Raman spectroscopic analysis. The white rectangle highlights the area captured in the bright-field images at the cartilage-bone interface. Raman spectra were recorded in the area with cell clusters and signs of cartilage calcification (*) in each image. Scale bar 100 µm.


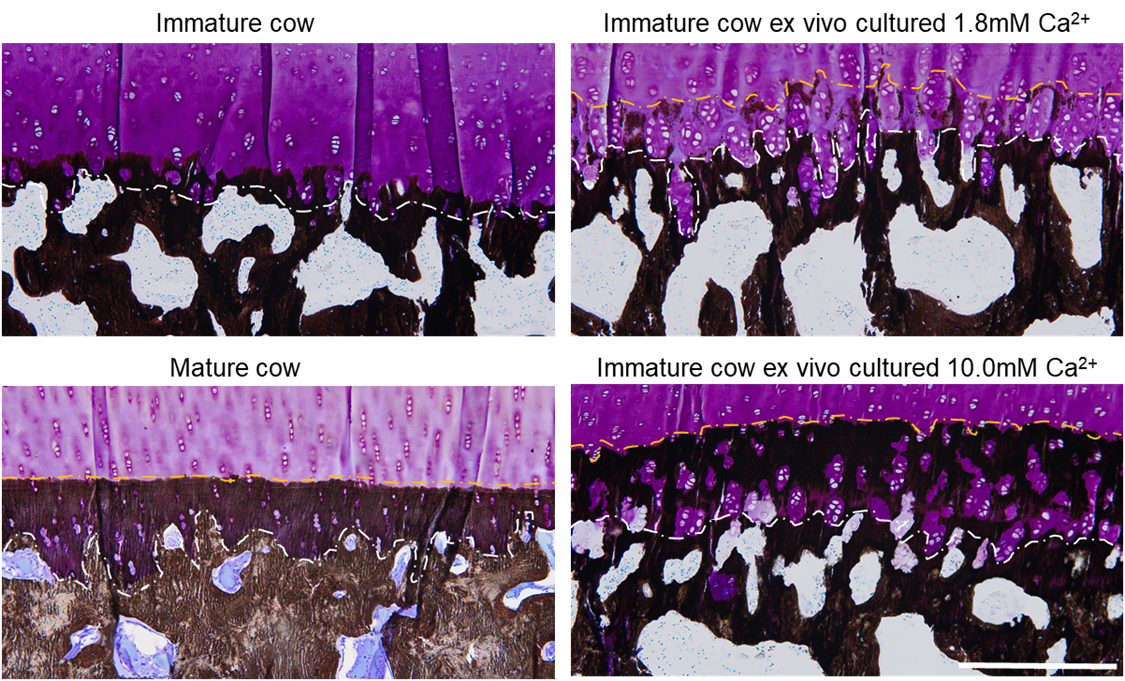


Supplementary Figure 4: Zoom in images of von Kossa stained osteochondral samples. In the freshly isolated sample of the immature cow, no zone of calcified cartilage (ZCC) is yet formed. The sample harvested from the mature cow shows the ZCC. First signs of the ZCC being formed in the immature samples after 17 days of ex vivo culture (media supplemented with β-glycerophosphate and 1.8mM or 10.0mM Ca^2+^) are shown in the representative samples on the right. Calcification of the formed non-calcified cartilage was more prominent in the sample culture at 10.0mM Ca^2+^ compared to 10.0mM Ca^2+^ concentration. The yellow line indicates the expected tidemark, the white line indicates the expected cement line. Scale bar 1mm.
